# Reappraising Abstract Paintings after Exposure to Background Information

**DOI:** 10.1371/journal.pone.0124159

**Published:** 2015-05-06

**Authors:** Seongmin A. Park, Kyongsik Yun, Jaeseung Jeong

**Affiliations:** 1 Graduate School of Culture Technology, Korea Advanced Institute of Science and Technology (KAIST), Daejeon, 305–701, Republic of Korea; 2 Department of Bio and Brain Engineering, Korea Advanced Institute of Science and Technology (KAIST), Daejeon, 305–701, Republic of Korea; 3 Centre de neurosciences cognitives (CNC) UMR 5229, Institut des Sciences Cognitives, CNRS, 67 Boulevard Pinel, 69675 Bron, France; 4 Computation and Neural Systems, Division of Biology, California Institute of Technology, Pasadena, California, 91125, United States of America; University College London, UNITED KINGDOM

## Abstract

Can knowledge help viewers when they appreciate an artwork? Experts’ judgments of the aesthetic value of a painting often differ from the estimates of naïve viewers, and this phenomenon is especially pronounced in the aesthetic judgment of abstract paintings. We compared the changes in aesthetic judgments of naïve viewers while they were progressively exposed to five pieces of background information. The participants were asked to report their aesthetic judgments of a given painting after each piece of information was presented. We found that commentaries by the artist and a critic significantly increased the subjective aesthetic ratings. Does knowledge enable experts to attend to the visual features in a painting and to link it to the evaluative conventions, thus potentially causing different aesthetic judgments? To investigate whether a specific pattern of attention is essential for the knowledge-based appreciation, we tracked the eye movements of subjects while viewing a painting with a commentary by the artist and with a commentary by a critic. We observed that critics’ commentaries directed the viewers’ attention to the visual components that were highly relevant to the presented commentary. However, attention to specific features of a painting was not necessary for increasing the subjective aesthetic judgment when the artists’ commentary was presented. Our results suggest that at least two different cognitive mechanisms may be involved in knowledge- guided aesthetic judgments while viewers reappraise a painting.

## Introduction

When we see an object, we can say whether it is beautiful and how beautiful it is. These questions are easy to answer, but it is difficult to answer the question how humans can make such aesthetic judgments. Identifying the factors that influence our aesthetic judgment is a good approach to answer that question. At first, the visual features do matter in aesthetic judgments. As many studies have demonstrated, symmetry, complexities, or composition, influence viewers’ aesthetic judgments [1–6]. Another factor that influences our aesthetic judgment is our knowledge about the object, which shapes our aesthetic valuation. For example, the auction price upsurges when bidders know the item is original or when it is associated with evaluative conventions such as personal history [7,8]. It is also very true that the value of artwork is not determined by its utility but varies according to the level of subjective knowledge [9–15]. Indeed, naïve viewers often hardly agree to the aesthetic judgments of art experts [16–18]. The impact of knowledge on the aesthetic judgment becomes more obvious when naïve viewers appreciate abstract paintings [12], whereby the artist’s intention is difficult to discern when compared to the intentions behind representative paintings. Likewise, the aesthetic judgment of abstract paintings tends to depend more heavily on the viewers’ knowledge [12].

Background information is typically provided to viewers who appreciate artwork in a gallery, a museum, or an auction house [19,20]. However, it remains unknown how the information influences the aesthetic judgment of the viewer while appreciating paintings. How does expertise lead to differences in aesthetic judgments? To address this question, it is necessary to examine how knowledge of an artwork guides the process of viewing works of art. In particular, using an eye-tracker allows researchers to track the attention processes of viewers, and recent studies have compared the processes of viewing visual artworks between experts and naïve viewers who had no prior knowledge of art. Several previous studies have suggested that different levels of knowledge or expertise differentiate viewers’ attention, with this potentially being the key to the different aesthetic judgments reached by naïve viewers and art experts [10,11,13,21–23]. However, it is difficult to identify which factors of previous knowledge are actually responsible for the specific pattern of attention that increases the aesthetic judgment of the works of art. In contrast, several studies have reported the significant impact of the provision of background information with regard to a work of art on the aesthetic judgments of naïve viewers [3–5,24]. However, the impact of the provided knowledge on changes in the process of appreciation, especially visual attention, has not been investigated sufficiently to date.

Neuroscientific findings suggest that perceptual processing by viewers may be essential for understanding how prior knowledge modulates changes in viewers’ aesthetic judgments. When prior information was available, perceptual decision-making required less effort than identical decisions made without any information [25–27], as demonstrated by the observation of reduced activity in brain regions associated with sensory processing when information is provided before viewing [25–27]. An EEG study found a similar effect of knowledge and extends this to the increases in the subjective value judgment of artworks, showing reduced gamma band activity in the left hemisphere of the brain of a viewer in association with increases in the subjective preference of artworks while viewing them [10].

Furthermore, background information can direct observers’ attention to the visual components in the artwork. If viewers enable to interpret those components in association with the subject of the painting or the unique characteristics of the artist, those evaluative interpretations may influence the subsequent aesthetic judgments. This information attracts viewers’ attention and influences their overall evaluations. Previous studies have suggested that differences in aesthetic judgments between art experts and naïve viewers result from differences in perceptions and memory retrieval during the appreciation of artwork [1,13,16,21,28–31].

Subjective interests determine the focus and duration of attention [1]. For example, in autism, the lack of social interest results in less attention being paid to social cues, whereas more social information processing occurs after instructing these individuals to pay attention to specific features [15,32,33]. The focus and duration of attention are significantly related to the evolution of preference. Recent studies demonstrated computational models supporting the influence of attention on evolving preferences. They demonstrated that the duration of attention predicts the preference-based choice between two alternatives [34–37], and a reciprocal relationship between attention and valuation [38,39].

In the present study, we examined whether increasing the viewer’s knowledge of a painting changes the subjective aesthetic valuation of an abstract painting. As the subjects were exposed to five pieces of information and repeatedly reappraised the artwork, we could compare the viewer’s initial aesthetic judgments with changes in attention after multiple reappraisals. Unlike previous studies that compared aesthetic judgments of experts with those of naïve viewers, we examine the changes in aesthetic judgments of naïve viewers while they reappraise artwork after learning background knowledge about the painting, as well as how their judgments differ from their initial aesthetic judgments made without any background information. Moreover, we performed another experiment to examine whether the specific patterns of attention are essential for higher appraisals during knowledge-based aesthetic appreciation of artworks. Using an eye-tracker, we compare the patterns of attention of viewers while they view the paintings after learning information that significantly influenced the behavioral responses of aesthetic judgments. We hypothesized that knowing the artist’s intention might increase familiarity with the artist by easing the viewers’ perceptual burden of interpreting the abstract expressions. In contrast, another type of information that provides specific information about spatial components of the painting may attract a viewer’s attention. When information emphasizes specific areas of the paintings and provides evaluative conventions attached to those areas, those visual properties subsequently attract the viewer’s attention during a reappraisal.

## Materials and Methods

### Subjects

The human subjects participating in these experiments were separated into two groups. First, 120 (116) subjects participated in a behavioral study (9–12 individuals per session, 65 females, mean age of 24.26 ± 2.79 years). Data acquired from 4 participants who studied fine art as their major in the university were excluded from the analysis. Second, 16 undergraduate students with normal vision participated in eye-tracking experiments (8 females, mean age of 23.84 ± 2.17 years). The participants were recruited through an advertisement on a local community website.

In this study, we focused on the effects of background information on the aesthetic value judgments of paintings. However, repetitive exposure in this paradigm would potentially affect aesthetic valuations (i.e., the mere-exposure effect) [40]. To reveal the effect of information on aesthetic valuations while the painting is reappraised and distinguish it from the potential effects of mere exposure (times of previous exposure to the painting), an additional behavioral experiment was conducted. Responses from 47 volunteer participants were collected via a webpage (29 females, mean age of 25.83±4.52 years).

We confirmed that no subject had a formal education in the visual arts, and data from individuals who were familiar with the painting or the artist were excluded from the analysis. In the self-reported questionnaire, no subjects indicated a history of neurological or psychiatric disorders. The participants received monetary compensation for completing the experiment. Informed written consent was obtained from all subjects, and the study was approved through the Institutional Review Board (IRB) of the Korea Advanced Institute of Science and Technology (KAIST) concerning human subjects and experimental procedures (KH2008-01).

### Stimuli

#### Selection of paintings

We selected eight experimental paintings based on the initial aesthetic judgments of 20 paintings obtained from another 258 subjects (117 females; mean age = 22.82 ± 6.96; 78.8% of these participants were in their twenties). The 20 abstract paintings were classified as “postwar and contemporary” artworks belonging to “a series” of work by an artist (one of multiple paintings created with the same intention by a single artist) for which an art auction trading record was set after the year 2000. We compared the levels of appreciation of the same painting between the initial exposure and a reappraisal after being exposed to each piece of information to minimize the potential influence of different initial aesthetic judgments among paintings on updating the aesthetic judgments upon the reappraisal. The subjects were asked to use a Likert scale ranging from 0–9 (9 = most preferred) to make aesthetic judgments based on the subjective aesthetic emotion generated while viewing the artwork without any cognitive information. The mean of the perception-based aesthetic valuations was normalized using the Likert scale. To select the paintings in which its aesthetic judgment was similar across viewers rather than the paintings in which both high and low aesthetic judgments coexist, we examined how much the aesthetic judgments of each painting were deviated from normality. We measured it as the Z value using the Kolmogorov-Smirnov test. Based on the subjective responses, we selected eight paintings with small variances from among 12 paintings (S.D < ±1.5) with relatively indifferent preferences (the Z-scores are in Table A in S2 File).

#### Background information

The following five different pieces of information were presented incrementally: “artist” included the artist’s name and the years of his birth and death; “title” included the painting’s title, the year when it was created, the materials used, and the size; the “artist’s commentary” was adapted from an interview or a note written by the painter expressing the general intention regarding the series of paintings; the “critic’s commentary” included a personal anecdote about the artist related to the painting or the meaning of depictions or expressions in the painting; and the “auction price” included the date of the most recent auction, the auction company, and the winning price. The length of each type of information was similar in Korean (see Tables B~H in S1 File).

#### Order of presentation of background information

The five pieces of information were presented in the same order among the subjects: artist, title, artist’s commentary, critic’s commentary, and the winning bid. The order of presentation was determined in advance because there is a semantic hierarchy among the pieces of information. The artist and the title of the painting are prerequisites to being exposed to the artist’s commentary. The critic’s comments assume that the viewer knows the artist’s intention concerning the series that includes the presented artwork; thus, the artist’s commentary takes precedence in the presentation order over the critic’s commentary. The winning bid information is presented last because this information was thought to have the potential to bias the following reappraisals if we provide it before presenting other information. In addition, the impact of different amount of the prices is also difficult to control across paintings. Thus, the order of presentation resembled the conventional order of information typically presented at an art gallery, museum, or auction house.

In this experimental design, our observations are limited to the influence of each type of information on the aesthetic judgments, and the investigation of potential changes in the accumulative effects is based on the presented order of the information. However, focusing on the impact of the fixed order of information, which has been used as a convention, facilitates an examination of the background information-based reappraisal process as distinguished from the initial viewing, which is subsequently influenced by the incremental effects of information on the subjects.

### Experimental design

#### Behavioral experiment

**Fig 1:**
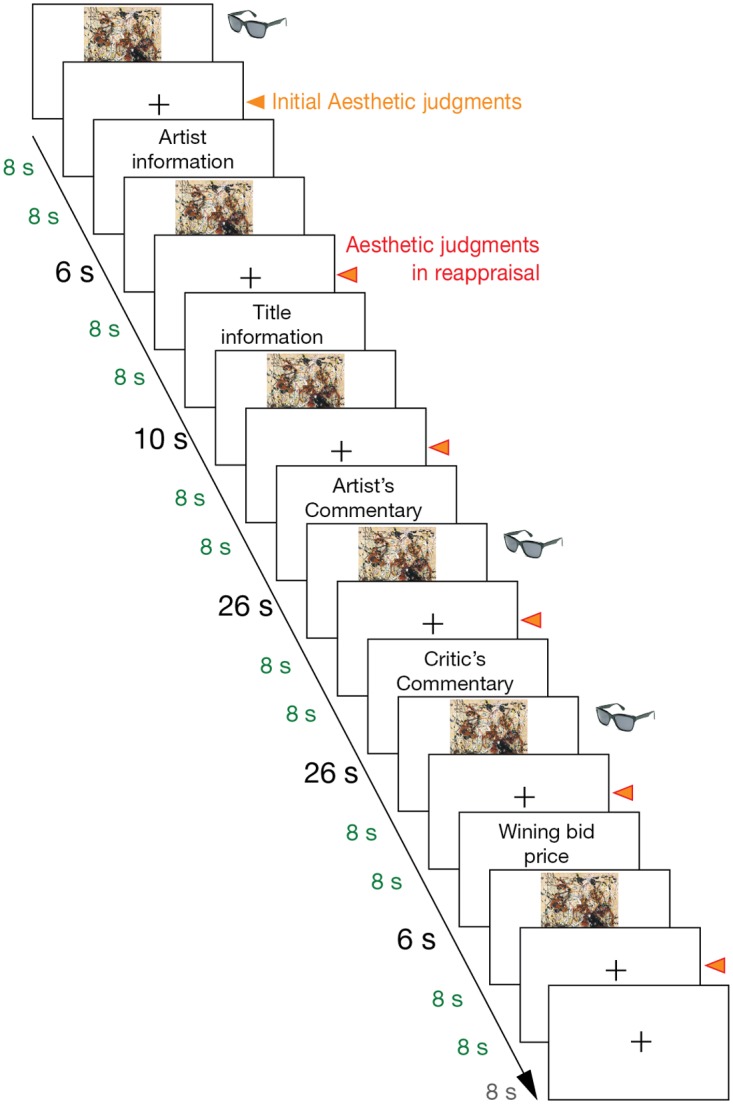
The timeline of a block of viewing experiences that includes the initial viewing and the five subsequent information-based reappraisals of a painting (*No*. *12* by Jackson Pollock in the figure). The painting was presented for 8 seconds (Green colored). A fixation cross was presented as the inter-stimulus interval between painting presentations. In the behavioral experiments, the subjects made their aesthetic judgments during the fixation at the indicated times, marked as triangles. The information was presented in the following order for a given duration: the artist information (6 seconds), the title information (10 seconds), the artist’s commentary (26 seconds), the critic’s commentary (26 seconds), and the most recent winning bid (6 seconds). The viewing of the next painting was begun after the inter-block interval (8 seconds). The perceptual patterns were acquired using an eye-tracker and were compared between modulations during the initial viewing and during the reappraisals after two types of commentaries (glasses symbol).

The subjects viewed the paintings for 8 seconds upon the initial exposure (without any cognitive information) and after being exposed to each piece of cognitive information, for a total of 6 viewings. A fixation point consisting of a stationary black cross was presented after each viewing (8 seconds). The information was presented in the following order and for the indicated times: the artist (6 seconds), the title (10 seconds), the artist’s commentary (26 seconds), the critic’s commentary (26 seconds), and the auction price (6 seconds). Prior to the experiment, a questionnaire was provided (Fig 1). The subjects were asked to view the painting for the full 8 seconds. Background information was then sequentially presented, and the increases in the reported aesthetic value of the painting and the reported meaningfulness of the painting based on their increased understanding of the painting were measured (see instruction in S1 File). An aesthetic judgment was made using a Likert scale (range: 1–9: 9 = most preferred). During the six fixation periods, the viewer made an aesthetic judgment of the painting and estimated the usefulness of, and agreement with, the provided information. The participants estimated the value of the information by rating “how the painting became meaningful after being exposed to each piece of information (usefulness)” and “agreement with the commentaries from the artists and critics” using a Likert scale (range: -2 ~ +2; +2 = very useful or highly agreeable). The familiarity was assessed after the last reappraisal of each painting. A bell sound was used at the end of the fixation period to attract the viewers’ attention to the screen. Detailed instructions about the “aesthetic judgments” and “meaningfulness” of the painting were provided prior to the experiments based on adaptations from a previous study [41,42]. An additional 8 seconds of fixation occurred after the processing of one painting had been completed and before the viewing of the next painting. The presentation order of the artworks was varied among the participant groups. The page of the questionnaire asking for the aesthetic valuation of a given artwork was separate from the pages used for the previous judgments, and the subjects did not need to remember their previous aesthetic judgments.

#### Eye-tracking experiment

Subjective attention, driven by the different pieces of information during the reappraisals, was compared with the attention patterns observed during the initial viewing. The subjects were asked to appreciate the same eight paintings, and their eye movements were tracked (Fig 1). The procedure was identical to that used for the behavioral assessment, with a few exceptions. The paintings were scaled down to preserve the original proportions and were fitted to a screen resolution of 1024 × 768 pixels. Fixation followed after viewing the paintings for 4 seconds. To control the starting point of attention and avoid potential distractions, the subjects were asked to gaze at the fixation cross (center of the screen) prior to viewing the paintings. The subjective value of the information was not estimated. The rating of an aesthetic judgment was acquired during fixation after viewing the painting using the same Likert scale used in the behavioral experiment. The subjects used a mouse to indicate their judgments. The familiarity-based recognition of each painting was surveyed after the experiment.

#### Behavioral experiment to control for the mere-exposure effect

To investigate whether the aesthetic value increased during repeated exposures to artworks, another separate group of subjects was asked to rate subjective aesthetic value changes while they viewed the paintings repeatedly (Fig 1). The same eight paintings that were presented in the behavioral experiment were presented in a pseudo-random order. First, four of the eight paintings were selected randomly by a computer and were presented six times in a predetermined order. To control the duration between the presentations of a painting, the order of each painting was determined; we replicated the presentation timing that had been used during the behavioral experiments. Second, the other four paintings were presented in the same predetermined order after a 10-second resting fixation. The participants were asked to make an aesthetic judgment to describe their subjective value of the paintings with the same Likert scale (range: 1–9: 9 = the most preferred) immediately after each appreciation during the presentation of a fixation cross. After a decision was made, the fixation cross was presented for an additional 2 to 5 seconds (jittered; see Fig. A in S1 File). The stimulus size was modulated with a consistent ratio according to the individuals’ screen sizes, as established before presenting the stimuli. Participants were instructed to respond using the number pad on their keyboard. After the series of aesthetic judgments was made, we asked the participants whether they had had any exposure to the paintings before the experiment.

### Data acquisition

To characterize the changes in the behavioral aesthetic judgments, the subjects viewed eight paintings at their actual size via a beam projector (NEC NP2000) on a white wall in an exhibition room at the Daejeon Museum of Art.

Next, the attention patterns influenced by the different commentaries were recorded and compared with the attention patterns from the initial viewing of each painting. We compared the attention patterns of each subject during the reappraisals, particularly after exposure to one of the two commentaries, with the initial viewing of the same painting using a Tobii T120 eye-tracker (Tobii Technology, Stockholm, Sweden) with a 120-Hz sampling rate. The luminance in the experimental room and the viewing distance (fixed at 60 cm) were maintained. After calibration, each artwork was presented at the center of the screen with a resolution of 1024 × 768 pixels on a white background. The spatial accuracy was better than 0.5°, and the drift was less than 0.3° in the pupil-tracking mode.

### Analyses

#### Behavioral data analysis

The influence of each type of information was estimated by increases in the aesthetic valuation compared with the judgment made during the prior viewing before being exposed to each piece of information. The significance level of the influence of the information was validated using repeated-measures ANOVAs. The aesthetic judgments of 8 paintings under the influence of each type of information were averaged for each subject across the subjects. Therefore, we compared changes in aesthetic judgments that were acquired at 6 times during the experiment. These included the initial judgment and changes in the judgments under the influence of 5 types of information. Furthermore, post hoc analyses were performed to examine whether the impact of each type of information significantly modulated the subjective aesthetic judgments. In addition, the relationship between the individual differences in the estimation of the usefulness of the information and the level of agreement with the commentaries and their influences (increases in aesthetic judgment) were investigated using correlation analyses.

#### Eye-tracking data analysis

To measure the spatial distribution of attention and the temporal dwelling time, the paintings were divided into a grid of square cells, and we acquired the eye-movement data from each unit area to investigate both spatial and temporal attention changes during viewing of the painting per painting using Tobii Studio 1.0 software (Tobii Technology AB, Danderyd, Sweden). The minimum size of the unit that humans can process as distinct visual information was calculated based on the size of the presented painting and the distance from the viewer and eye-tracker in our experiment [43]. The attention time for a grand cell was the average duration of attention for nine adjoining small-grid cells. The attention time of each grand cell was acquired from each subject three times: during the first appreciation (no information), during the appreciation after exposure to the artist’s comment, and during the appreciation after exposure to the critic’s comment. By comparing attentional durations in each grand cell within the same appreciation time, 8 seconds, we investigated the influence of the two types of comments on attention for each cell among the subjects. Considering the repeated measures, we compared the degree of attention using a repeated measures ANOVA using SPSS (SPSS, IBM, Somers, NY). Three attention time values were used as the input in the repeated measures calculations. Because the different comments may correspond with different areas of each painting, we could not compare the overall effect of one type of commentary on the paintings overall. Instead, we performed separate analyses for each cell for each of the eight paintings. The aim of this analysis was not to compare the impacts of the two commentaries with regard to how they modulated attention on the same area of paintings but to investigate what type of commentary attracts a viewer’s attention to a specific area of a painting and whether this modulation could be observed among all participants. As such, we investigated both the main effects of times and post hoc comparisons between the amounts of attention after exposure to one of the commentaries and during the initial appreciation. The Bonferroni method was used to adjust for multiple comparisons (p-value < 0.05; see the number of samples in Table B in S2 File).

## Results

### Behavioral results

The average initial aesthetic judgment for the presented works of art was 5.11 ± 0.061 (standard error of the mean (*SEM)*). This perception-based aesthetic judgment increased to 5.159 ± 0.059 upon a reappraisal with the artist information. When presented with the title information, the aesthetic valuation increased to 5.340 ± 0.057. The aesthetic valuation of artwork followed by the artist’s commentary was appraised as 5.686 ± 0.059 and increased to 6.034 ± 0.058 when the critic’s commentary was presented. Finally, the aesthetic valuation of the artwork reached a score of 6.17 ± 0.060 after the participants knew the recent bid information, i.e., when all of the information about the artwork was made available.

**Fig 2:**
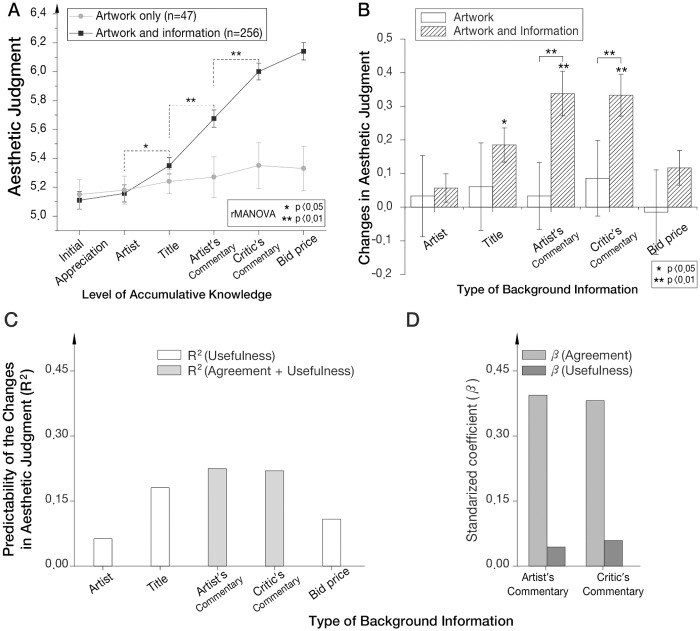
Behavioral changes in aesthetic judgment as a function of the background information. (A) Sequential changes in self-reported aesthetic judgments through the incremental learning of background information. (B) Influence of each type of background information. Each bar denotes an increase in aesthetic judgment compared to the judgment made prior to being exposed to each piece of information. The changes in aesthetic judgment after being exposed to the title information, the artist’s commentary, and the critic’s commentary were significant (repeated measures ANOVA; *: *P* < 0.05; and **: *P* < 0.01). (C) The correlation coefficient (R-squared) between the subjective assessment of each type of background information and changes in aesthetic judgments (*P* < 0.001). (D) The value of the standardized coefficient β indicates the power of the increases in the agreement with (light gray) and usefulness of (gray) the commentary to predict increases in aesthetic judgments (multiple regression analysis; *P* < 0.001). The error bars represent the standard error of the mean (SEM).

The presented information increased the participant’s aesthetic valuations during the iterated viewing task. Sphericity-assumed modeling with the Greenhouse–Geisser adjustment was applied (ε = 0.477). Fig 2A shows the sequential increase in aesthetic valuation upon the acquisition of each type of information (F (2.385:253.061) = 285.149, p-value = 6.750 × 10–5). Using Bonferroni post hoc analyses, we observed that the knowledge of the artist and both commentaries significantly increased the aesthetic valuations compared to the judgments made prior to receiving each type of information (p-value < 0.05). For the two commentaries, we observed a significant cubic effect at the threshold p-value = 8.284 x 10–15 (F (1:115) = 74.382). This result indicated that there were two instances of significant increases in aesthetic judgment induced by the two types of commentaries, supporting the effects of those two types of background information on the reappraisal of aesthetic judgments. We also observed a significant fourth-order effect of the title information and the two commentaries at the threshold p-value = 0.021 (F (1:115) = 7.381). This effect indicated that there were three significant increases in aesthetic judgment, those induced by the title information, artist’s commentary and critic’s commentary, supporting the effects of those three types of background information on the reappraisal of aesthetic judgments (Fig 2A).

When the same eight paintings were appreciated without background information, the initial aesthetic valuation was 5.155 ± 0.103 (SEM), which was not significantly different from the initial judgment of the participants who appreciated the paintings with background information. The aesthetic value was 5.184 ± 0.097 after the second exposure of these paintings, 5.243 ± 0.083 after the third exposure, 5.274 ± 0.141 after the fourth exposure, and 5.359 ± 0.158 after the fifth exposure. The final aesthetic valuation was reported as 5.331 ± 0.154 (Fig 2A).

Fig 2B illustrates the changes in the aesthetic judgments as a function of the type of information presented, indicating the influence of the information on the aesthetic judgments of the contemporary abstract paintings. These data indicate that the commentaries from the artists and critics were more influential than the other pieces of information in the participants’ increased aesthetic valuation. The effects of repetitive exposures were tested by repeated measures ANOVAs; no significant changes in aesthetic valuations due to the effect of repeated appreciation trials without background information (p-value > 0.05) were observed. We also compared the differences in aesthetic valuations due to each type of information and the differences in aesthetic valuations due to the effects of repeated exposures. The increases in aesthetic value after exposure to the artist’s commentary were significantly greater than the changes due to the three exposures (p-value = 3.243×10^-8^), and the increases in aesthetic value after exposure to the critic’s commentary were significantly greater than the changes due to the four exposures (p-value = 6.692×10^-6^) (Fig 2B).

The computed R^2^ values correlating the meaningfulness of the paintings and the changes in aesthetic judgments compared to the judgments before each type of information was presented were as follows: artist, 0.063; title, 0.181; and the most recent and highest bid information, 0.108 (Fig 2C, p-value < 0.001).

We also conducted multiple regression analyses for the commentary information and observed a significant correlation among the aesthetic judgment changes, the usefulness of the commentary, and the degree of agreement. The regression model estimates the aesthetic judgment change upon being exposed to the artist’s commentary, showing R^2^ values of 22.5% by 0.394 × Agreement + 0.044 × Usefulness − 1.097. The aesthetic judgment change upon being exposed to the critic’s commentary showed R^2^ values of 22.0% by 0.382 × Agreement + 0.059 × Usefulness − 1.244. The standardized coefficient beta confirmed that the influence of agreement was stronger than that of the perceived meaningfulness of the commentary information (Fig 2D, p-value < 0.001).

### Eye-tracking results

**Fig 3:**
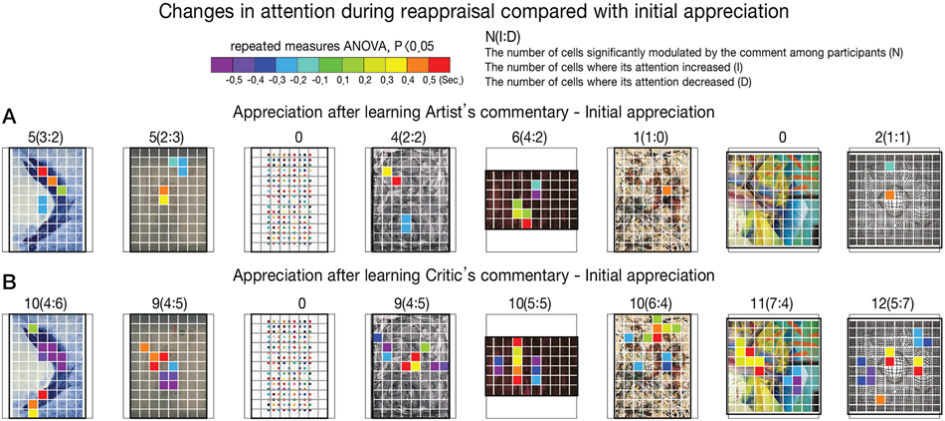
The changes in duration of attention after (A) the artist’s commentary and (B) the critic’s commentary. Each cell indicates the size of the unit used for the analysis of attention at both spatial and temporal levels. We colored the cell area of the painting that showed significant changes in attention across the participants (repeated measures ANOVA, P<0.05). The color indicates the amounts of increases or decreases in attention on the cell.

We hypothesized that despite the significant impact of both the artist’s and critic’s commentaries on increases in the aesthetic valuations, only the critic’s commentary would require the voluntary attention of viewers to conform to the critic’s aesthetic judgments. To test our hypothesis, the number of grid cells in each painting that showed significant differences in the duration of attention was calculated. The difference between the initial attention and the modulated attention after exposure to each type of commentaries made by an artist and a critic was calculated for each individual and for each painting. We observed that more cells showed significant attention duration differences among the participants when reappraising the artwork under the influence of the critic’s commentary than when reappraising after the artist’s commentary. Based on the repeated measures ANOVAs and post hoc comparisons with the amount of attention during the initial appreciation, the number of grand cells that showed significant modulations in attention due to one or both commentaries and their positions are illustrated in Fig 3. The color of the heat map in Fig 3 indicates the amount of significant change in the attention time, including both increases and decreases (repeated measures ANOVA, p-value <0.05). We also report the epsilon in Mauchly's test of sphericity as corrected by Greenhouse-Geisser, the significance level, and the amount of modulation in attention with standard deviations (Table B in S2 File).

## Discussion

Compared with perception-based artwork appreciation and its aesthetic judgments, little is known about cognitive knowledge-based reappraisal and the underlying mechanisms of increases in aesthetic valuation. In the present study, we assessed the aesthetic valuations of naïve viewers during repeated reappraisals of abstract paintings. To facilitate naïve viewers’ aesthetic appreciation of the paintings, we progressively provided five pieces of cognitive information, as conventionally provided by galleries. Recent neuroimaging studies suggest that the cognitive process of making aesthetic judgments includes emotional processing, attention, and decision-making. Viewers who have prior knowledge about an artwork show different attention patterns and brain activations, especially during visual information processing [11,13,16,21,32]. A viewer’s attention, the locus and the duration until making a decision are significantly related to the preference-based choices [35,37,44–46]. In this study, we examined the relationship between attention as modulated by cognitive information and changes in aesthetic judgments.

We observed that background information, particularly commentaries by the artist and by a critic, significantly increases subjective aesthetic valuation, which was also significant when we compared these increases with the increases in the subjective aesthetic judgments due to mere-exposure effects. The effects of exposure to these commentaries on increases in the aesthetic valuations of artwork were also significant when we compared them with the mere-exposure effect as measured from reports by a different participant group on increases in their preferences upon repeated exposure to the same visual stimuli. We found significant correlations between the increases in meaningfulness of the paintings and the amount of agreement with the commentaries and with the changes in aesthetic judgment when the commentaries were available. Thus, the aesthetic valuations increased when the provided information makes the painting more meaningful to the viewers, and the amount of modulation during the aesthetic judgment depends on the level of subjective agreement with the commentaries from experts. This may occur because abstract paintings are likely less easily familiarized when the viewer cannot understand their meaning, or it may be due to the boredom of the participants, which is known to be a limiting factor of the mere-exposure effect [43].

We also compared the reappraisal process with the initial viewing at an attentional level. We demonstrate that knowledge of the background information increases aesthetic pleasure through changes in attention processes, suggesting two different types of attention modulation through information. The different patterns in attention were modulated through the two different commentaries. We observed that fewer areas of the painting showed significant attention modulation after exposure to the artist’s commentary than after exposure to the critic’s commentary. These analyses included both increases and decreases in the amount of attention compared to the initial appreciation, supporting the notion that participants' interest levels change as a result of the provided commentaries.

In this study, we presented artwork from an artist’s series created under a specific theme. When the viewers were given the artist’s commentary, they learned the artist’s original intention and what he tried to express through the artwork series. Unlike the critic’s commentary, an artist’s commentary increased the aesthetic valuation, but did not attract any relevant attention patterns across the subjects. We speculate that the reasons may underlie such increases in aesthetic judgment by the artist’s commentary. Though these are highly realted with each other, we listed them in three based on previous literatures. First, it may help the viewer to understand the artist’s intent and to distinguish meaningful figures from abstract forms; viewers also tend to like what they know, which is accompanied by easier visual information processing [47–49]. Second, the knowledge also may increase the familiarity of the viewer with the presented artwork; indeed, the influence of familiarity on increases in aesthetic valuation has been well investigated [2,50–53]. Third, this phenomenon could be caused by enhanced cognitive mastery at the level of intrinsic motivation of viewers to search for future exposure to the artwork. Such mastery has been suggested to increase interest in artwork over the long term [11]. These speculations are supported by previous studies suggesting that foreknowledge decreases the computational burden of visual perception by complementing visual input [47–49] and that the preceding interpretation of sensory information is reconciled when visual stimuli appear [54,55].

However, more attention was directed to specific parts of a painting during a reappraisal after being exposed to the critic’s commentary. Unlike the artist’s commentary, the critic’s commentary requires viewers to detect specific visual components in the painting not previously attended to by the viewers. Cognitive information can increase the visual salience of contextually relevant targets and modulate subsequent stimulus-driven processes during subsequent viewings [3,24,56,57]. A previous study has reported brain attention network responses to a more preferred style of representational paintings that have indeterminate elements [47]. Having access to a third person’s perspective, such as a critic’s perspective, may lead to voluntary attention, which simulates the valuation processes of others. During the reappraisal, compared to the initial viewing, the background information guides the viewer’s perception, increasing the understanding of ambiguous abstract expressions of the artist’s intention or modulating voluntary attention processes to focus on newly updated points of interest related to specific parts of the painting.

Does the specific content of the critic’s comments lead the viewers to attend to a specific part of a painting that corresponds to the context? Compared with the artist’s commentaries, which covered the common theme of the series of artworks, the critic, focusing more on the specific painting presented to the viewers, could include the meaning of specific painting expressions and could discuss why the painting was important within the artist’s career. For example, we found that the viewers directed their attention to ‘the vertical existential line’ in the Newman painting when the critic mentioned this element. We speculate that the viewers reconcile the symbolic expression with the concept of a ‘cry of humankind to God’ or that the critic’s commentary ‘brings to life’ the painting during the viewing after exposure to the commentary. We found that the viewers followed the trace of the movement of a female model in the Klein painting after they had received information related to this via the critic’s commentary, in this case ‘engaging models to wander, naked, around the studio while he painted’. We also speculate that the viewers may combine this action with what the commentary includes about the meaning of the color blue: ‘sensuousness and life’. The viewers may simulate the movement of the painter after they know how the Pollock painting was created, and the action gained in aesthetic value to the viewers who agreed with the critic’s perspective on the ‘action of drawing’. Where the viewers focused on the Vasarely painting was on the variation forms of black squares, and this attention may stem from the critic’s phrase ‘copying themselves and moving forward with little changes, making us a spatial and mobile illusion’, and ‘time is stopped, but an accident is repeated infinitely’. Taken together, we speculate that the specific parts of paintings that received increased attention had high semantic congruency with the critic’s commentary.

The results of this study should be understood while considering the following limitations. The five types of background information were presented in a fixed order in this study. To investigate the independent influence of each type of information, further study is needed. Repeated appreciation trials can increase the subjective value of an artwork with increases in familiarity but can also decrease it, possibly due to increases in boredom. These effects can be modulated by differences in individuals and artworks. Because we cannot investigate the effects of exposure to information upon reappraisals except with the potential effect of multiple appreciations of the same artworks, we performed an additional experiment and compared both effects. Despite the significant increases in aesthetic value after exposure to information compared to the increases due to the mere-exposure effect, we cannot exclude the possible effect of repeated appreciation trials on the reported cognitive process associated with aesthetic value changes. In addition, we did not show the impact of each type of information separately on the aesthetic judgments. This distinction is difficult because the information presented to the viewers has a hierarchical association (as it does in conventional galleries).

Our results show that the newly given cognitive information is critical to improve naïve viewer’s aesthetic judgments in abstract painting. Higher evaluations after a critic's commentary were accompanied by changes in attention, while those after the artist's commentary were not. It suggest at least two different cognitive mechanisms may be involved in knowledge-guided aesthetic judgments while viewers re-appraise a painting. To the best of our knowledge, this study is the first investigation of the influence of different information types on sequential changes in aesthetic judgments within the same group of participants. Further studies are needed to reveal the influence of specific information on aesthetic judgments and the connections between how this information influences attention and the computation of the value of the artwork in the brain.

## Supporting Information

S1 File Supporting information of materials and methods. It shows the introduction that we gave to the subjects for understanding of the experiment, and the stimuli (both paintings and background information).
(PDF)

S2 File Supporting information about results. It includes the statistical results for experimental painting selection, and the modulations in attention under influences of two different commentaries.
(PDF)
