# Supplementary material for: Reappraising Abstract Paintings after Exposure to Background Information
Source: PLoS One. 2015 May 6;10(5):e0124159. doi: 10.1371/journal.pone.0124159 (PMC4422661; doi:10.1371/journal.pone.0124159)
Supplement: S1 File — It shows the introduction that we gave to the subjects for understanding of the experiment, and the stimuli (both paintings and background information). (PDF) [file pone.0124159.s001.pdf]

Supplementary Information S1

Materials and Methods

Reappraising abstract paintings after exposure to background information

Seongmin A. Park, Kyongsik Yun, and Jaeseung Jeong

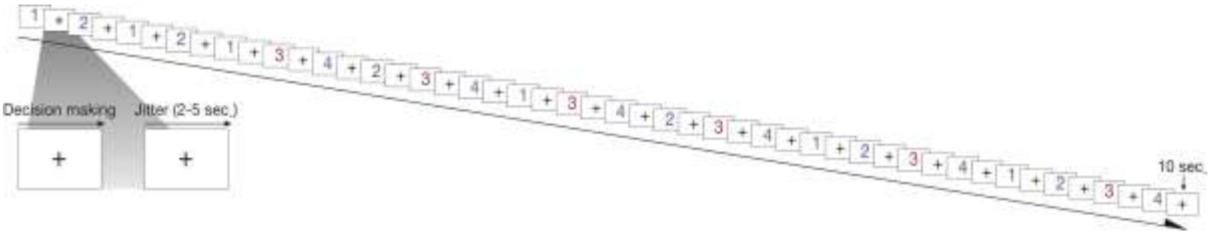

Figure 1 in S1 File. The experimental procedures to investigate the mere exposure effect on the aesthetic value of artworks. 4 among 8 paintings that were selected randomly by a computer were presented 6 times in the predetermined order. To control the duration between presentations of a painting, the order of painting was decided: we attempted to replicate the similar presentation timing that had been used during the behavioral experiments'. Second, the other 4 painting was presented in the same predetermined order, after the 10 seconds of fixed fixation. The participants were asked to make an aesthetic judgment to describe their subjective value of the paintings with the same Likert scale (range: 1 – 9: 9 = most preferred) right after each appreciation during the presentation of fixation cross. After a decision was made, a fixation cross continued for 2 to 5 seconds.

Supplementary Information S1

Materials and Methods

Instructions

Prior to the experiments, participants were fully informed about the concept of aesthetic appraisal [1-3] with the following instructions:

> Aesthetic judgment is based on the amount of artwork-specific aesthetic emotions generated by the intrinsic qualities of the presented artwork. Positive aesthetic emotions may be simplified as joy, thrill, awe, being moved, and enjoyment of a beautiful, well-structured or intensely expressive artwork, whereas negative aesthetic emotions may be simplified as boredom, disgust, annoyance, or disinterest in a blatant and ugly piece of artwork. In making an aesthetic appraisal decision, try to compare the painting with other paintings you have encountered in your life (on a 10-point scale; 0 - 9; 9 indicates the most preferred artwork). There is no right or wrong answer. We are simply interested in your personal opinion. You do not need to remember your previous aesthetic appraisal of the identical painting.

The instructions on meaningfulness [4,5], which were used to assess the usefulness of the information, were also presented prior to the experiments as follows:

> How meaningful is the presented painting to you after learning the information provided immediately prior to seeing the artwork? The meaningfulness is the extent to which you are able to understand the

Materials and Methods

painting, make sense of it, and see what it intends to represent. In coming to a decision about the usefulness of the information, try to consider how the painting becomes meaningful to you when presented with the background information (on a 5-point scale; 1 - 5: very useful for appreciating the painting). There is no right or wrong answer. We are simply interested in your personal opinion.

Stimuli

Paintings and Background information presented in the experiment

To select paintings for the experiment from 12 candidate contemporary abstract paintings, impression-based aesthetic appraisal was first determined via a web-based survey on a 7-point scale. In total, 258 people (117 females; mean age of 22.8 years) without expertise in fine-art were asked to provide their impressions of the presented paintings based on their gut reaction. Data from 19 participants who knew of the painting were excluded from the analyses. Except for the visual stimuli, no other cognitive information was provided. According to the responses, 8 paintings that were rated at mid-level preference with small individual differences between responses were selected for the experiment. Table S1 shows the candidate paintings and the responses of the perception-based aesthetic appraisal. In gallery experiments, participants were asked to fully appreciate the artwork for 4 s, and a bell rang to turn their attention from the questionnaires to the artwork so that the duration of appreciation among the participants was controlled.

Supplementary Information S1

Materials and Methods

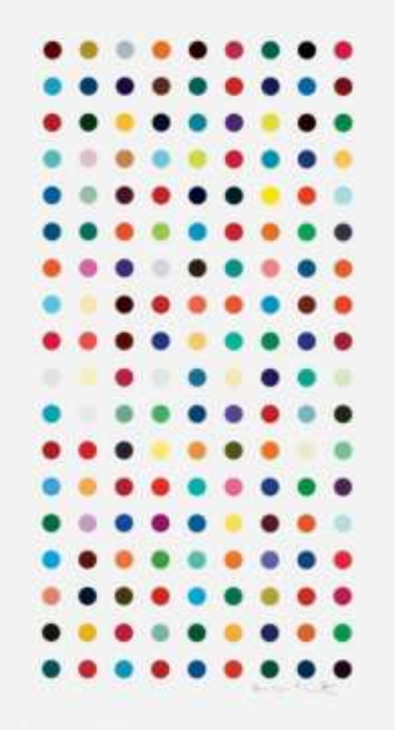

Information 1: Artist	Damien Hirst (1965 -)
Information 2: Title	Methamphetamine (2004)<br>Etching printed in colors, 2004, signed in pencil recto, numbered 52/115, published by Paragon Press, on woven paper, 177 x 86.5 cm [6]
Information 3: Artist's commentary	Art is like medicine - it can heal. Yet I've always been amazed at how many people believe in medicine, but don't believe in art, without questioning either. In the spot paintings the grid-like structure creates a beginning of a system. On each painting no two colours are the same. This ends the system; it's a simple system. No matter how I feel as an artist or a painter, the paintings end up looking happy. I can still make all the emotional decisions about colour that I need to as an artist, but in the end they are lost. The end of painting, and I'm still painting and all art should be uplifting for a viewer. I love colour. I feel it inside me. It gives me a buzz. I hate taste - it's acquired. [7].
Information 4: Critic's commentary	The subject of this spot series by Hirst, known as a mischievous boy in the UK, came from nervous narcotic drugs. It is another addiction to find beauty, which is the only purpose when appreciating the paintings. The beauty in previous art came from mythic stories that neglect the reality and threaten us with something that's solely beautiful to fix our artistic sensibilities. But as we see with this painting, the sum of the minimum elements of paintings (color, shape) can create beauty by itself. Indeed, paintings can include more than just the beautiful; through this painting, we might believe that people who want to find unreal beauty depend on drugs and in previous paintings neglect the real world [6].
Information 5: Recent winning bid price	Estimate: $20,000 - 30,000 [6]

Table 1 in S1 File. A painting and its background information presenting to the viewers

Supplementary Information S1

Materials and Methods

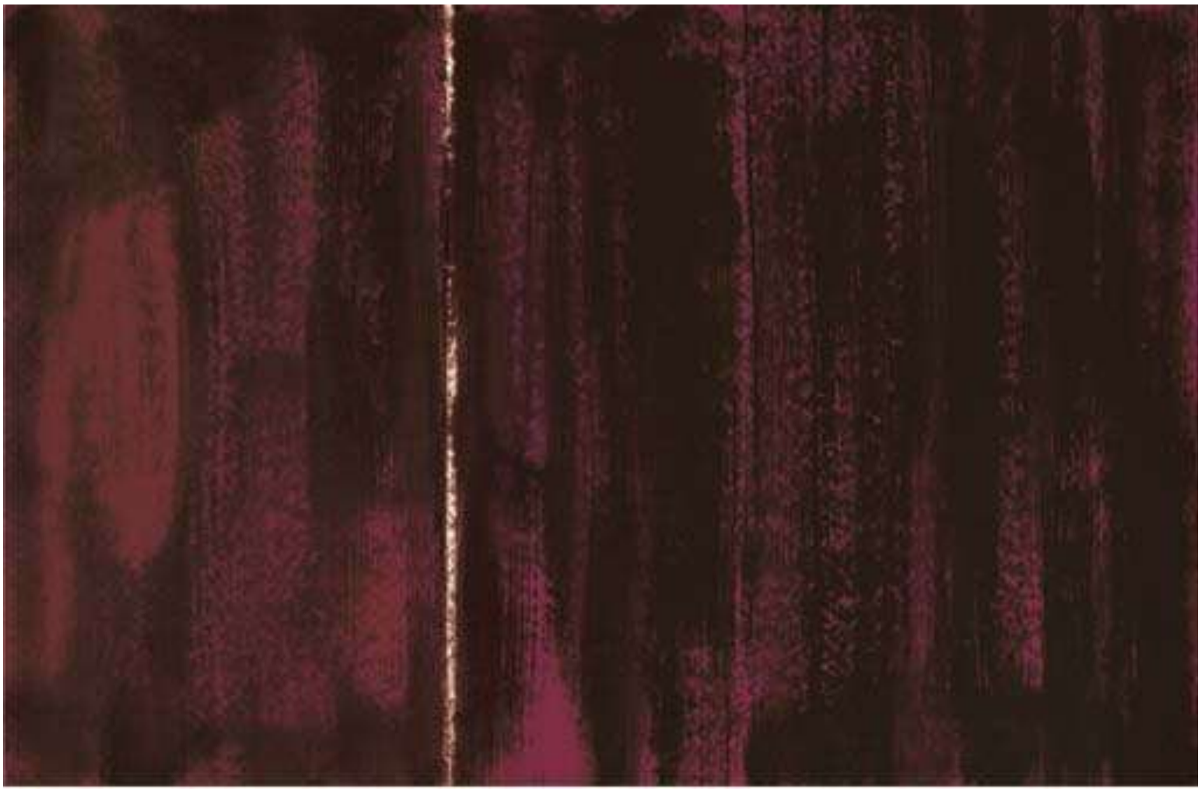

Information 1: Artist	Barnett Newman (1905 – 1970)
Information 2: Title	Untitled 2 (1948)<br>signed, titled and dated '"Untitled" 1948 Barnett Newman' (on the stretcher)<br>oil on paper mounted on canvas<br>24 3/8 x 36 3/8 in. (61.8 x 92.4 cm.)<br>Painted in 1948.
Information 3: Artist's commentary	I feel that kind of physical suffering has gotten almost universal. I was trying to call attention to that part of the passion which I have always felt was ignored and which has always affected me. That was the cry "Lema Sabachthani (why have you forsaken me)," which I don't think was a complaint, but which Jesus makes. I was always stuck by the paradox that of those who actually persecuted him and crucified him, he says to God, "Forgive theme, for they know not what they do," but to God, who is projected as his father, he says, "What's the idea?" This to me is a very strange paradox. In doing this work I began to feel hat my paintings were making that kind of a statement about the cry. … This is what the paintings meant to me – the cry.[8]
Information 4: Critic's commentary	*Untitled 2* is one of only two oil paintings made on paper by Newman that exist, and both of these works belong to the period immediately after *Onement I* …This vertical existential line, signified for Newman man's physical and existential nature amidst a metaphysical void. … The union or integration of this single existential line with the apparently infinite field of the background was what led to him later entitling the work and others like it *Onement*. … For Newman, the 'zip' was not a line but a 'field' in the same way that his backgrounds can be considered 'fields'. An integral and necessary part of the whole it was a field that "brings to life other fields, just as other fields bring to life this so-called line [9]
Information 5: Recent winning bid price	$1,024,000 [9]

Table 2 in S1 File. A painting and its background information presenting to the viewers

Supplementary Information S1

Materials and Methods

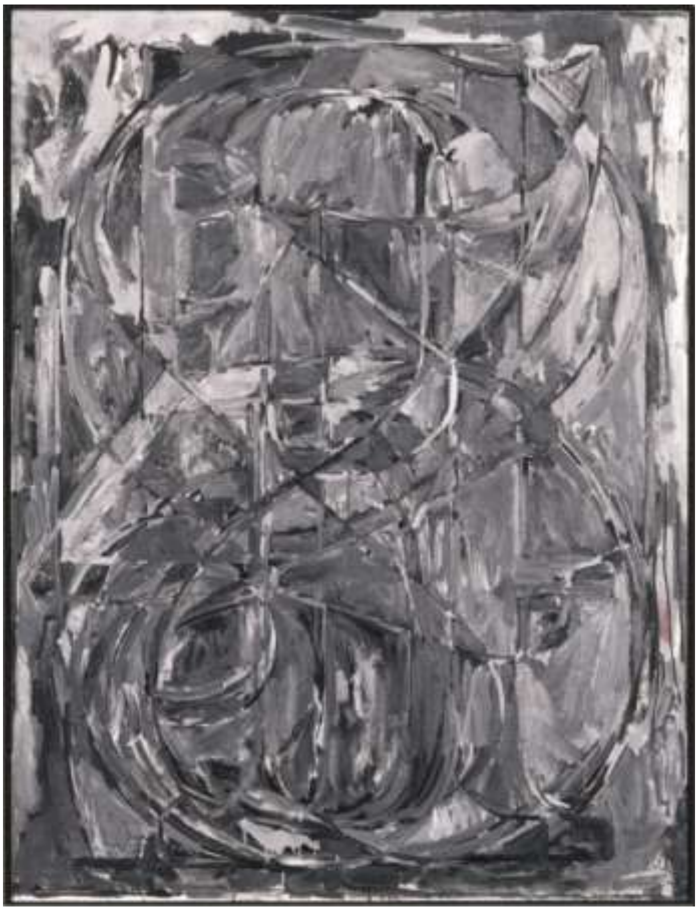

Information 1: Artist	Jasper Johns (1888-1976)
Information 2: Title	0 through 9 (1961)<br>Encaustic on canvas<br>137.1 x 105.4 cm
Information 3: Artist's commentary	I'm interested in things which suggest the world rather than suggest the personality." Johns maintained, "I'm interested in things which suggest things which are rather than in judgments. The most conventional things, the most ordinary things - it seems to me that those things can be dealt with without having to judge them; they seem to me to exist as clear facts not involving hierarchy [10].
Information 4: Critic's commentary	Beginning with the number '0' and working through the sequence to the point where the painting of number "9" completes the painting, this process allows Johns to build up the painting through a series of progressive and pre-determined stages. At each stage, Johns is forced to make a certain number of additions and yet at the same time is free - due to the increasing abstraction of the image - to make a number of aesthetic decisions of his own. The process of painting has become an interactive game between the artist and his subject matter, and in the end, it is the nature of the game and of the process of painting that the finished work emphasizes rather than its systematic structure or beginnings. Through the process of being made the painting becomes something more than the sum of its parts. It becomes not only a visual record of its own creation but also an enigma, a manifestation of the mystery of art and the act of creation. [6].
Information 5: Recent winning bid price	$9,909,500 [6]

Table 3 in S1 File. A painting and its background information presenting to the viewers

Supplementary Information S1

Materials and Methods

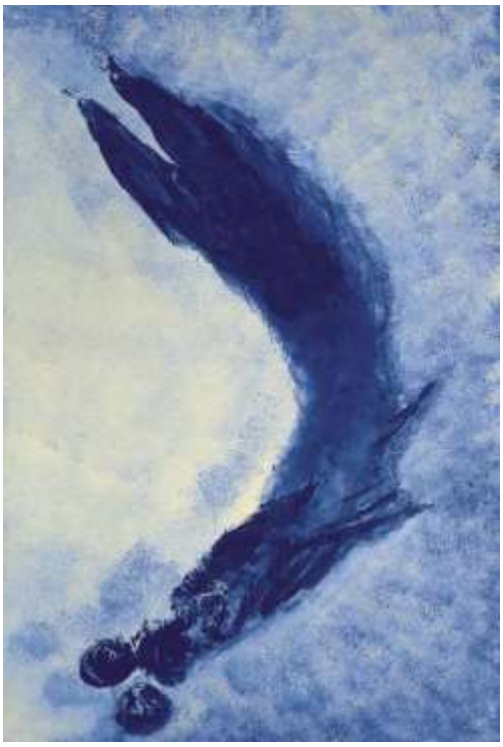

Information 1: Artist	Yves Klein 1928-1962
Information 2: Title	ANT 127 (Anthropometries series 127) (1960)<br>signed and dated 'Yves Klein 1960' (on the reverse side) pigment (IKB: International Klein Blue: artist's own patented color) on paper laid on canvas<br>225 x 145 cm
Information 3: Artist's Commentary	My models laughed more than a little when they saw how I created the exquisite blue monochrome, limited to one color, after their images! They laughed, but they felt more and more attracted to the blue. One day it was clear to me that my hands and tools were no longer sufficient to work with the color. I needed the model to paint the monochrome painting. … That was, finally, the solution to the problems of distance in painting: My brushes were alive and remote-controlled. … I stayed clean. I no longer dirtied myself with color, not even the tips of my fingers. The work finished itself there in front of me, under my direction, in absolute collaboration with the model. And I could salute its birth into the tangible world, in a dignified manner, dressed in a tuxedo [11].
Information 4: Critic's Commentary	When Klein was creating his monochrome works in this infinite IKB, he invoked an air of sensuousness and of life (All the better to invoke the spirit of the immaterial and to capture it in the material world) by engaging models to wander, naked, around the studio while he painted. …Intriguingly, Klein's interest in the naked female form had become specific during the creation of the Anthropometries, it is not the complete body, but rather the headless and limbless torso that was the focus of his work, as is clear in Ant 127. For Klein, this emphasized the raw power of life. He has removed intellect from his pictorial equation, and instead has focused on the necessity-driven, animal of the human body [11].
Information 5: Recent winning bid price	$4,048,000 [11]

Table 4 in S1 File. A painting and its background information presenting to the viewers

Supplementary Information S1

Materials and Methods

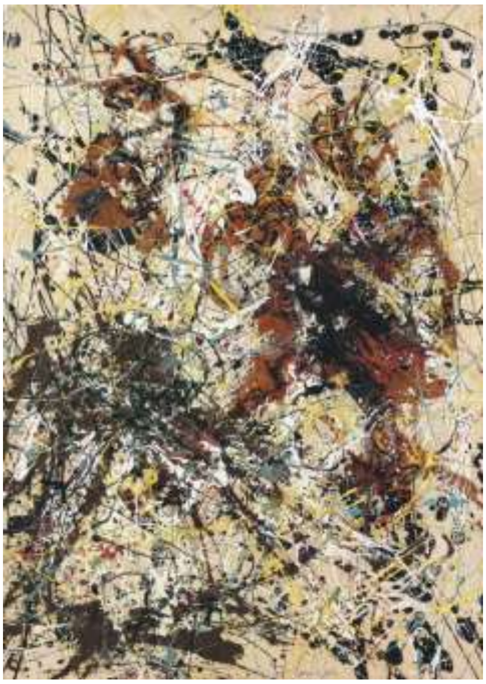

Information 1: Artist	Jackson Pollock (1912 – 1956)
Information 2: Title	Number 12 (1949)<br>oil on paper laid down on masonite, signed and dated 'Jackson Pollock 49' (lower center) 78.8 x 57.1 cm [12]
Information 3: Artist's commentary	The modern artist is living in a mechanical age and we have a mechanical means of representing objects in nature such as the camera and photograph. The modern artist, it seems to me, is working and expressing the inner world- in other words- expressing the energy, the motion, and other inner forces … the modern artist working with space and time, and expressing his feelings rather than illustrating<br><br>When I am in my painting, I'm not aware of what I'm doing. It is only after a sort of 'get acquainted' period that I see what I have been about. I have no fears about making changes, destroying the image, etc., because the painting has a life of its own. I try to let it come through. It is only when I lose contact with the painting that the result is a mess. Otherwise there is pure harmony, an easy give and take, and the painting comes out well [13,14].
Information 4: Critic's commentary	After World War II and the great economic recession, fine art could not exist as representing the beauty of nature while ignoring the suffering in the real world. The world did not feel beautiful anymore, and nature became distant from humankind. The paintings, losing their model to paint, maintained the action: only draw. Therefore, every moment of the painter's action became the only and the most genuine and sincere thing. …<br>Pollock breaks the previous concept of art with every fiber of his body and develops the technique of dropping paint to express his inner mind; thus began abstract expressionism [15,16].
Information 5: Recent winning bid price	$11,655,500 [12]

Table 5 in S1 File. A painting and its background information presenting to the viewers

Supplementary Information S1

Materials and Methods

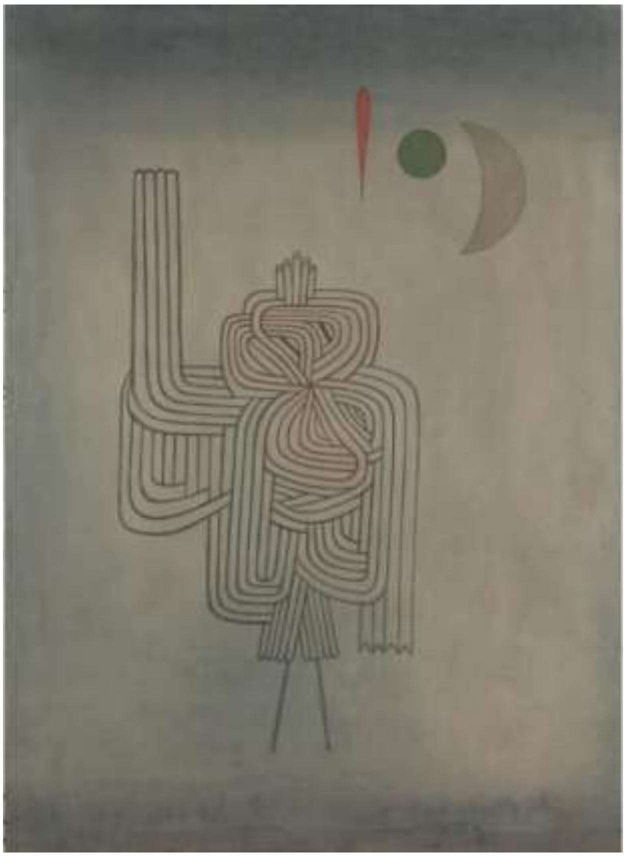

Information 1: Artist	Paul Klee (1879 - 1940)
Information 2: Subject	Gespenster Abgang (Departure of the ghost) (1931)<br>Watercolor, gouache, and pen and ink on muslin mounted on canvas<br>96 x 70.5 cm [17]
Information 3: Artist's commentary	Art is a likeness of the Creation. Occasionally, it is an example, just as the terrestrial may exemplify the cosmic. Art does not reproduce the visible; rather, it makes visible…. I cannot be grasped in the here and now. For I reside just as much with the dead as with the unborn. Somewhat closer to the heart of creation than usual. … In the final analysis, a drawing simply is no longer a drawing, no matter how self-sufficient its execution may be. It is a symbol, and the more profoundly the imaginary lines of projection meet higher dimensions, the better [16,18].
Information 4: Critic's commentary	Paintings can represent the visible existence, but the camera is a better media for that. Klee realized the possible existence somewhere of an imaginary universe, and he expresses that on the canvas. At first, his paintings look abstract because of their unfamiliarity, but after understanding the subjects and diving into his world, objects and his intentions become clear. … Though he has excellent *dessin* technique, most of his paintings express the innocent. Including imaginative power, a child-like point of view, harmony of colors, and the making of sound visual, his paintings express the root of genuineness [17].
Information 5: Recent winning bid price	$2,256,000 [17]

Table 6 in S1 File. A painting and its background information presenting to the viewers

Supplementary Information S1

Materials and Methods

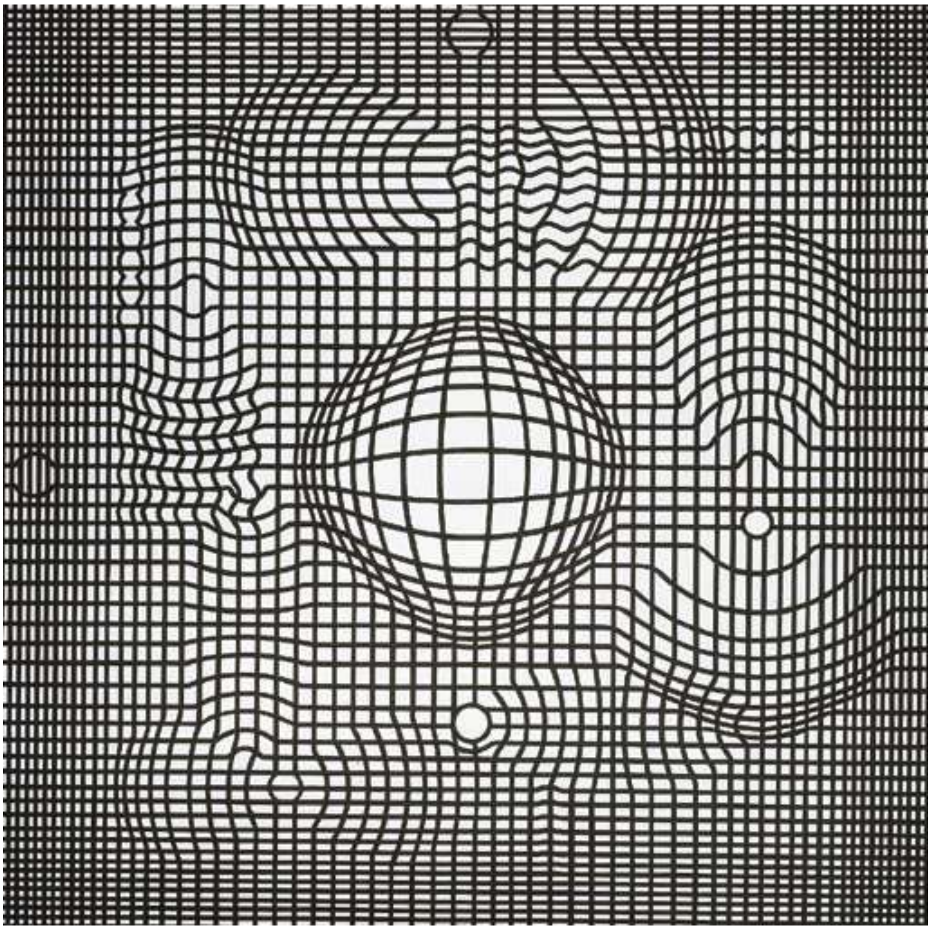

Information 1: Artist	Victor Vasarely (1906 - 1997)
Information 2: Subject	Jone (Jong) (1962)<br>oil on canvas<br>205.7 x 205.7 cm [6]
Information 3: Artist's commentary	The maximum contrast is Black and White... Negatives-positives, transparencies, photograms, the hallucinatory play of black and white I deduced there from, philosophically speaking, that white and black signs, the inevitable antinomy of the ideas of the past, like "day and night", "angel and devil", "good and evil", are in reality complementarities, a fertile androgynous idea. Coupling affirmation and negation in unity is making knowledge integral. What a prospect! White and black, it is the indestructibility of art-thought and hence the perenniality of the work in its original form[19].
Information 4: Critic's commentary	A white square with a black border that already has all of the elements for painting. The basic form of the painting was formed from another entire painting by copying themselves and moving forward with little changes, making us a spatial and mobile illusion. … His painting is like a digital world that is a simple thing, which anyone can make, and it becomes a whole new thing by being copied and collected. In his painting, time is stopped, but an accident is repeated infinitely [6].
Information 5: Recent winning bid price	$296,000 [6]

Table 7 in S1 File. A painting and its background information presenting to the viewers

Supplementary Information S1

Materials and Methods

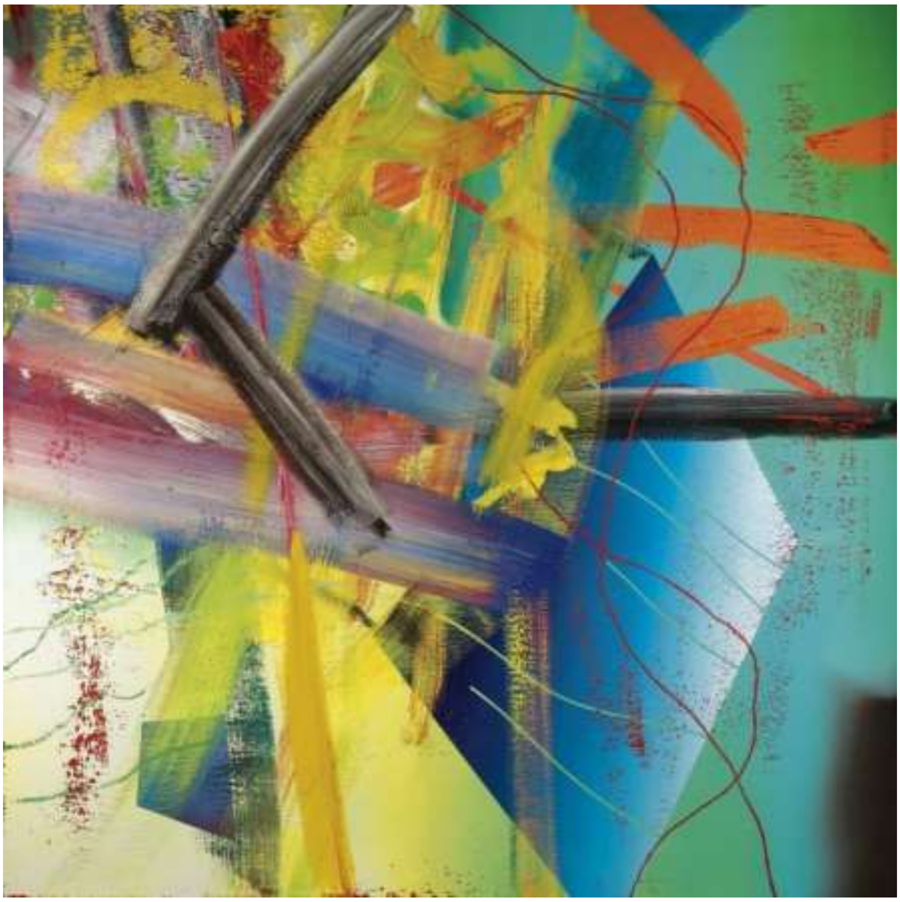

Infomation1: Artist	Gehard Richter (1932 -)
Infomation2: Subject	Maria (1983)<br>on the reverse<br>200 x 200 cm [20]
Information 3: Artist's commentary	Every time we describe an event, add up a column of figures or take a photograph of a tree, we create a model.... Abstract paintings are fictitious models because they visualize a reality which we can neither see nor describe but which we may nevertheless conclude exists. We attach negative names to this reality, the un-known, the un-graspable, the infinite, and for thousands of years we have developed it in terms of substitute images like heaven and hell, gods and devils [21].
Information 4: Critic's commentary	For Richter, the art of painting is both a deeply problematic and moral obligation. Aside from being a compulsive channel for personal expression, it is a means for him to explore the easily overlooked concepts of perception, ideology and belief through which we construct the world surrounding us. Like his photo-based paintings which underline the role of pictures in understanding the fictive nature of reality, his abstract paintings similarly avoid and confront problems of representation simultaneously. They instantaneously expose their own artificiality and weaknesses, offering approximations of reality which are enigmatically familiar yet ones we can never truly comprehend [22]
Information 5: The most recent winning bid price	$2,368,000 [20]

Table 8 in S1 File. A painting and its background information presenting to the viewers

Supplementary Information S1

Materials and Methods

References

1. Brattico E, Jacobsen T (2009) Subjective Appraisal of Music. Annals of the New York Academy of Sciences 1169: 308-317.
2. Rozin A, Rozin P (2008) Feelings and the enjoyment of music. Behavioral and Brain Sciences 31: 593-594.
3. Strawson P (2004) Aesthetic appraisal and works of art. Aesthetics and the Philosophy of Art: The Analytic Tradition: an Anthology.
4. Martindale C, Moore K, Borkum J (1990) Aesthetic preference: Anomalous findings for Berlyne's psychobiological theory. The American journal of psychology 103: 53-80.
5. Russell PA (2003) Effort after meaning and the hedonic value of paintings. British Journal of Psychology 94: 99-110.
6. Christie's (2003) Auction Catalogue: Post-War and Contemporary art, November 2003. In: Christie's, editor. Christie's.
7. Hirst D, Burn G, Morgan S, Barnbrook J, Violette R (1998) I want to spend the rest of my life everywhere, with everyone, one to one, always, forever, now: Booth-Clibborn.
8. Newman B, O'Neill JP (1992) Barnett Newman: selected writings and interviews: Univ of California Pr.
9. Christie's (2005) Auction Catalogue: Post-War and Contemporary art, May 2005. In: Christie's, editor. Christie's.
10. Francis R, Johns J (1984) Jasper Johns: Abbeville Pr.
11. Christie's (2006) Auction Catalogue: Post War and Contemporary Art (Evening Sale), 2006. In: Christie's, editor. Christie's.
12. Christie's (2004) Auction Catalogue: Post War and Contemporary Art (Evening Sale), 2004. In: Christie's, editor. Christie's.
13. Ross C (1990) Abstract expressionism: creators and critics: an anthology: Harry N Abrams Inc.
14. Namuth H, Rose B (1980) Pollock painting: Dodd Mead.
15. Guggenheim Online database of Jackson Pollock in Guggenheim museum.
16. Ruhrberg K (2000) Art of the 20th Century: Taschen America Llc.
17. Sotheby's (2006) Auction Record, "Impressionist & Modern Art Evening" (Auction Dates: Session 1: Tue, 07 November 06 7:00 PM). In: Sotheby's, editor. Sotheby's.
18. Guggenheim Online database of Paul Klee in Guggenheim museum.
19. Joray M Vasarely: Plastic Arts Of the 20th Century. Editions Du Griffon Neuchatel,(Switzer.
20. Sotheby's (2006) Auction Record, "Contemporary Art" (Auction Dates:14 November, 2006 7:00 PM). In: Sotheby's, editor. Sotheby's.
21. Richter G (1982) Exhibition Catalogue, Documenta 7: Gerhard Richter.
22. Angelika Thill ea (1993) Gerhard Richter Catalogue Raisonné 1962-1993, Ostfildern-Ruit, Vol. III.
