# Supplementary material for: Reappraising Abstract Paintings after Exposure to Background Information
Source: PLoS One. 2015 May 6;10(5):e0124159. doi: 10.1371/journal.pone.0124159 (PMC4422661; doi:10.1371/journal.pone.0124159)
Supplement: S2 File — It includes the statistical results for experimental painting selection, and the modulations in attention under influences of two different commentaries. (PDF) [file pone.0124159.s002.pdf]

Reappraising abstract paintings after exposure to background information

Seongmin A. Park, Kyongsik Yun, and Jaeseung Jeong

Painter	Title of painting	Normalized mean aesthetic appraisal (0-1)	Individual difference (Kolmogorov-Smirrnov Z)
Willem De Kooning	Untitled XVII	0.520	5.097
Hans Hartung	T1957-13	0.467	4.574
Yves Klein	Anthropometries series 127	0.466	3.622
Paul Klee	Gespenster Abgang	0.454	3.207
Jackson Pollock	Number 12	0.439	2.901
Jasper Jones	0 through 9	0.436	2.962
Damien Hirst	Methamphetamine	0.431	3.207
Gehard Richiter	Maria	0.430	2.851
Victor Vasarelly	Jong (Jone)	0.427	3.013
Barnett Newman	The Word Ⅱ	0.410	2.916
Josef Albers	Study for homage to the square: Looking ahead	0.389	3.712
Ellsworth Kelly	Yellow curve	0.381	3.708

Table A in S2. Candidate paintings and the subject's responses for the perception-based aesthetic appraisal.

Supplementary Information S2 Results

Paintings							
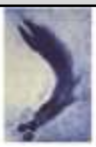	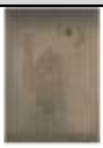	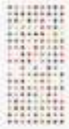	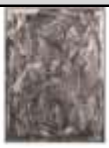	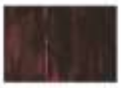	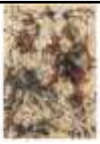	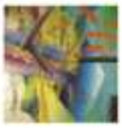	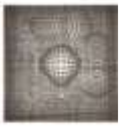
**Attention modulated by Artist's commentary**							
0.560±0.083<br>0.031<br>0.41	0.420±0.020<br>0.022<br>0.77		0.610±0.065<br>0.012<br>0.57	0.560±0.090<br>0.006<br>0.42	0.410±0.099<br>0.023<br>0.51		0.480±0.062<br>0.014<br>0.33
0.420±0.100<br>0.018<br>0.47	0.350±0.080<br>0.009<br>0.6		0.350±0.073<br>0.043<br>0.47	0.290±0.028<br>0.01<br>0.51			-0.140±0.054<br>0.013<br>0.67
0.130±0.024<br>0.018<br>0.22	-0.170±0.053<br>0.011<br>0.6		-0.210±0.029<br>0.044<br>0.66	0.180±0.032<br>0.033<br>0.26			
-0.260±0.025<br>0.048<br>0.65	-0.240±0.033<br>0.02<br>0.62		-0.220±0.017<br>0.042<br>0.53	0.150±0.047<br>0.041<br>0.26			
-0.270±0.081<br>0.041<br>0.61	-0.240±0.030<br>0.01<br>0.39			-0.140±0.030<br>0.009<br>0.77			
				-0.530±0.052<br>0.03<br>0.42			
**Number of cells showing significant differences**							
5(3:2)	5(2:3)	0	4(2:2)	6(4:2)	1(1:0)	0	2(1:1)

Attention modulated by Critic's commentary							
0.400±0.090<br>0.031<br>0.34	0.590±0.084<br>0.034<br>0.57		0.610±0.092<br>0.026<br>0.59	0.720±0.088<br>0.015<br>0.68	0.700±0.092<br>0.018<br>0.69	0.520±0.064<br>0.021<br>0.62	0.690±0.047<br>0.044<br>0.66
0.340±0.044<br>0.017<br>0.75	0.540±0.070<br>0.011<br>0.63		0.560±0.039<br>0.037<br>0.75	0.710±0.058<br>0.026<br>0.54	0.450±0.065<br>0.032<br>0.28	0.380±0.053<br>0.012<br>0.6	0.540±0.097<br>0.009<br>0.39
0.250±0.020<br>0.017<br>0.26	0.490±0.075<br>0.026<br>0.63		0.370±0.079<br>0.043<br>0.65	0.400±0.026<br>0.009<br>0.25	0.200±0.081<br>0.014<br>0.69	0.360±0.073<br>0.022<br>0.36	0.440±0.071<br>0.048<br>0.22
0.180±0.066<br>0.006<br>0.45	0.470±0.080<br>0.031<br>0.3		0.140±0.018<br>0.008<br>0.39	0.360±0.075<br>0.02<br>0.24	0.190±0.047<br>0.022<br>0.23	0.350±0.029<br>0.043<br>0.38	0.370±0.085<br>0.025<br>0.79
-0.450±0.029<br>0.048<br>0.43	-0.340±0.016<br>0.048<br>0.41		-0.220±0.035<br>0.033<br>0.47	0.230±0.077<br>0.006<br>0.36	0.180±0.098<br>0.026<br>0.74	0.340±0.079<br>0.007<br>0.77	0.340±0.068<br>0.03<br>0.52
-0.530±0.028<br>0.024<br>0.47	-0.460±0.076<br>0.02<br>0.42		-0.340±0.058<br>0.044<br>0.41	-0.230±0.031<br>0.033<br>0.28	0.130±0.018<br>0.011<br>0.56	0.290±0.017<br>0.045<br>0.25	-0.200±0.052<br>0.024<br>0.5
-0.540±0.084<br>0.04<br>0.51	-0.550±0.025<br>0.038<br>0.75		-0.490±0.087<br>0.027<br>0.59	-0.380±0.088<br>0.009<br>0.68	-0.220±0.070<br>0.036<br>0.27	0.270±0.031<br>0.04<br>0.24	-0.270±0.075<br>0.019<br>0.28
-0.600±0.057<br>0.037<br>0.29	-0.560±0.048<br>0.008<br>0.24		-0.530±0.093<br>0.008<br>0.49	-0.390±0.095<br>0.037<br>0.64	-0.230±0.093<br>0.032<br>0.51	-0.240±0.027<br>0.042<br>0.45	-0.320±0.064<br>0.044<br>0.54
-0.610±0.060<br>0.033<br>0.37	-0.670±0.095<br>0.037<br>0.23		-0.540±0.030<br>0.009<br>0.76	-0.510±0.035<br>0.038<br>0.67	-0.270±0.039<br>0.025<br>0.72	-0.250±0.027<br>0.039<br>0.55	-0.380±0.061<br>0.01<br>0.52
-0.670±0.076<br>0.038				-0.540±0.097<br>0.02	-0.340±0.028<br>0.026	-0.440±0.096<br>0.043	-0.390±0.058<br>0.046

0.24				0.63	0.66	0.78	0.42
						-0.580±0.031	-0.440±0.016
						0.041	0.048
						0.56	0.71
							-0.480±0.056
							0.006
							0.75
**Number of cells showing significant differences**							
10(4:6)	9(4:5)	0	9(4:5)	10(5:5)	10(6:4)	11(7:4)	12(5:7)

Table B in S2. Reported the number of cells where its attention was modulated significantly during reappraisal of a painting after learning artist commentary or critic's commentary compared to attention on the same painting at initial appreciation using the repeated measures ANOVA and post-hoc analyses that the Bonnferonni correction was applied for the adjustment for multiple comparisons (In association with the Figure 3). The first row indicates the changes in attention duration (second; the decreases in attention marked in red), the second row indicates the significant level of this modulation (*P* value < 0.05), and the third row indicates the epsilon (ε) as the results of the sphericity test for the repeated measures ANOVA that was corrected by the Greenhouse-Geisser methods.
